# Supplementary material for: Evolution of A bHLH Interaction Motif
Source: Int J Mol Sci. 2021 Jan 5;22(1):447. doi: 10.3390/ijms22010447 (PMC7794824; doi:10.3390/ijms22010447)
Supplement: Supplementary file 1 [file ijms-22-00447-s001.zip › Fig S1_dis-pred_idds.pdf]

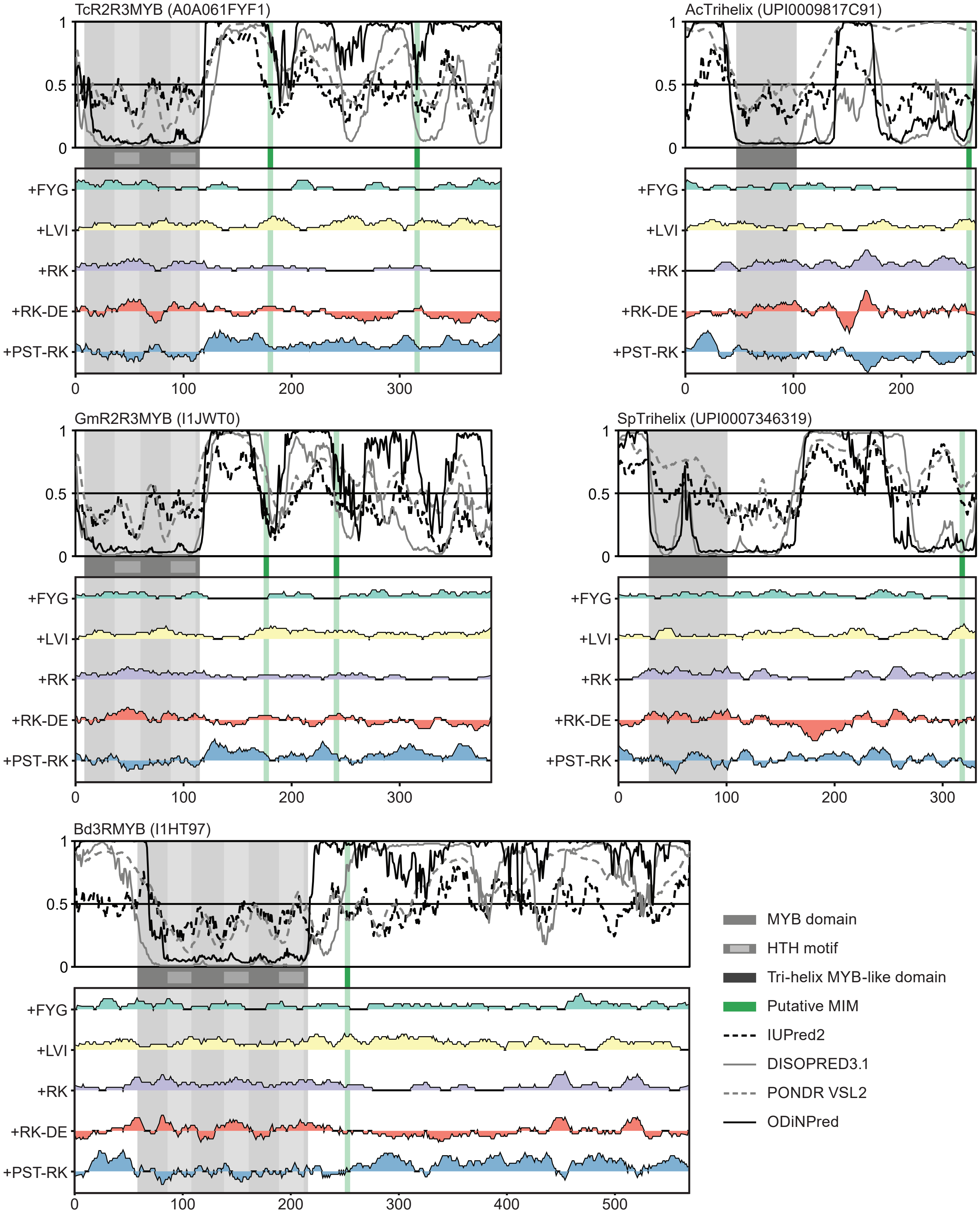

Supplemental Figure 1. Disorder prediction and IDDomainSpotter profiles for putative MIM containing proteins. Top panels show sequence specific disorder predictions using IUPred2 [49], DISOPRED3.1 [50], PONDR VSL2 [51,52], and ODiNPred [53]. Bottom panels show IDDomainSpotter profiles: Each curve shows the fraction of a different set of residues within sliding windows of 15 residues [48]. Domain information was obtained from PROSITE [41]. The putative motif is shown by a green bar.
