# Supplementary material for: Evolution of A bHLH Interaction Motif
Source: Int J Mol Sci. 2021 Jan 5;22(1):447. doi: 10.3390/ijms22010447 (PMC7794824; doi:10.3390/ijms22010447)
Supplement: Supplementary file 1 [file ijms-22-00447-s001.zip › Fig S2_Y2H_.pdf]

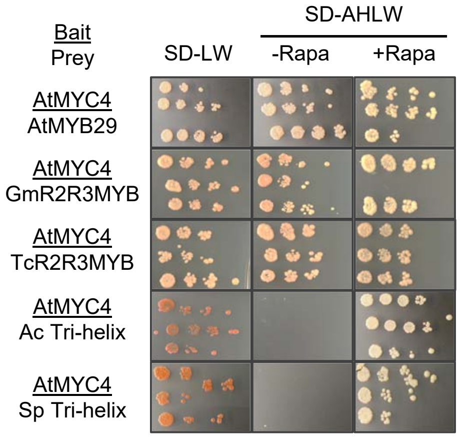

Supplemental Figure 2. Testing interaction between *A. thaliana* (At) MYC4 against AtMYB29, MIM-containing R2R3 MYB TFs from *Glycine max* (GmR2R3MYB) and *Theobroma cacao* (TcR2R3MYB), and MIM-containing tri-helix MYB-like TFs from *Ananas comosus* (Ac Tri-helix) and *Solanum pennellii* (Sp Tri-helix). Growth on SD-LW medium confirms successful co-transformation with bait and prey construct and growth on SD-AHLW+Rapa medium indicates that bait and prey proteins are expressed and present in the same cellular compartment. The SD-AHLW-Rapa medium is selective for physical interaction of bait and prey protein. Rapa, rapamycin.
