# Supplementary material for: Evolution of A bHLH Interaction Motif
Source: Int J Mol Sci. 2021 Jan 5;22(1):447. doi: 10.3390/ijms22010447 (PMC7794824; doi:10.3390/ijms22010447)
Supplement: Supplementary file 1 [file ijms-22-00447-s001.zip › Fig S3_McMYC-droptest.pdf]

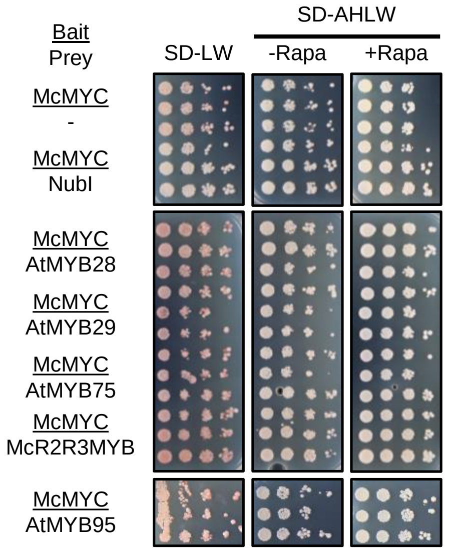

Supplemental Figure 3. Testing interaction between *Macleaya cordata* (Mc) MYC against empty prey plasmid (-), positive control (NubI), AtMYB28, AtMYB29, AtMYB75, AtMYB95 and MIM-containing R2R3 MYB TF from *M. cordata* (McR2R3MYB). Growth on SD-LW medium confirms successful co-transformation with bait and prey construct and growth on SD-AHLW+Rapa medium indicates that bait and prey proteins are expressed and present in the same cellular compartment. The SD-AHLW-Rapa medium is selective for physical interaction of bait and prey protein. Rapa, rapamycin.
