# Supplementary material for: Evolution of A bHLH Interaction Motif
Source: Int J Mol Sci. 2021 Jan 5;22(1):447. doi: 10.3390/ijms22010447 (PMC7794824; doi:10.3390/ijms22010447)
Supplement: Supplementary file 1 [file ijms-22-00447-s001.zip › Table S1_PSSMSearch.docx]

**Supplemental Table 1.** PSSMSearch results using an alignment of the MIM-containing region from the eight MIM-containing *Arabidopsis thaliana* R2R3 MYB TFs as query. The LN and A in the core motif xLNxxA were set as required, and the search was restricted to *A. thaliana* with default parameters and disorder cut-off at 0.4. The results were filtered by localization (nucleus, nuclear part, nucleoplasm part, nuclear transcription factor complex, nucleoplasm, nuclear transcriptional repressor complex) and warnings (topology, disorder, surface accessibility, localisation, domain).

| **#** | **Protein Name** | **GO term (localization filter)** | **Sequence** | **Start** | **End** | **PSSM P-value** | **QFO** | **Disorder score** |
| --- | --- | --- | --- | --- | --- | --- | --- | --- |
| 1 | Transcription factor MYB122 | nucleus | NKKLSGSSSARFLNRVANRFGHRINHNV | 159 | 186 | 1.1e-08 | 1.000 | 0.43 |
| 2 | Protein HUA2-LIKE 2 | nucleus | SDPSHGRDESTLLNEDASAAEQMLALRD | 154 | 181 | 3.7e-06 | 1.000 | 0.65 |
| 3 | Ethylene-responsive transcription factor ABI4 | nucleus | GSGCNNNSSMEDLNSLAGSVGSSLSITH | 247 | 274 | 4.0e-06 | 1.000 | 0.505 |
| 4 | Transcription factor MYB3R-1 | nucleus | DSPCHDRSPDVMLNDTAKSFSGAPSILK | 621 | 648 | 7.9e-06 | 1.000 | 0.436 |
| 5 | Nuclear pore complex protein NUP58 | nuclear part | TSNPSSGAGFSFLNTPASGPSSSLFATP | 409 | 436 | 1.6e-05 | 1.000 | 0.566 |
| 6 | Probable serine/threonine-protein kinase WNK4 | nucleus | SQSSSHSGSYSNLNYIAVDEYSSQSPVM | 448 | 475 | 1.6e-05 | 1.000 | 0.434 |
| 7 | FBD-associated F-box protein At3g49020 | nucleus | EELEKLGKRREMLNELATQASPSNSCNL | 415 | 442 | 2.7e-05 | 1.000 | 0.496 |
| 8 | RNA polymerase II C-terminal domain phosphatase-like 2 | nuclear part nucleus nucleoplasm part | NEGMCGGEVERRLNQAAAADHSTLPATS | 446 | 473 | 2.7e-05 | 1.000 | 0.594 |
| 9 | Ankyrin repeat domain-containing protein 2A | nucleus | ASGSSTSSAMPGLNFNAFDFSNMASILN | 30 | 57 | 2.7e-05 | 1.000 | 0.418 |
| 10 | Ubiquitin carboxyl-terminal hydrolase 24 | nucleus | KDKCVGSIQFGSLNLAAENSSVNTNGEL | 33 | 60 | 3.1e-05 | 1.000 | 0.464 |
| 11 | Histone-lysine N-methyltransferase MEDEA | nuclear part nucleus | SDSVIGKRQIYYLNGEALELSSEEDEED | 151 | 178 | 4.0e-05 | 1.000 | 0.429 |
| 12 | GATA transcription factor 25 | nucleus | DDGGSVADAANNLNTEAASVEEHTSMVS | 269 | 296 | 5.1e-05 | 1.000 | 0.534 |
| 13 | Protein TPX2 | nucleoplasm nuclear part nucleus | GSHQKSVKGETNLNNIASTTNLIQENQA | 202 | 229 | 5.2e-05 | 1.000 | 0.567 |
| 14 | Zinc-finger homeodomain protein 10 | nucleus | NNNNNNLASFSDLNFSAGNNHHHHHQHT | 168 | 195 | 5.2e-05 | 1.000 | 0.499 |
| 15 | Transcription factor MYC1 | nucleus | EDLHYKRTISTVLNYSADRSGKNDKNIR | 250 | 277 | 5.7e-05 | 1.000 | 0.443 |
| 16 | B3 domain-containing protein At3g19184 | nucleus | VEENKKRMGELNLNKLAQSLRVSSSSSS | 16 | 43 | 5.8e-05 | 1.000 | 0.498 |
| 17 | Transcription factor bHLH110 | nucleus | SVSLSHNYNNEMLNTRAHNNNNNNNTSE | 51 | 78 | 6.8e-05 | 1.000 | 0.494 |
| 18 | Zinc finger CCCH domain-containing protein 43 | nucleus | SGDSSHDRSLSDLNHAAEDLSDKLKNVG | 21 | 48 | 6.9e-05 | 1.000 | 0.528 |
| 19 | Uncharacterized protein At1g21580 | nucleoplasm part nuclear part | NVSGKGYMETTPLNVAAETADNMDSEEG | 792 | 819 | 6.9e-05 | 1.000 | 0.543 |
| 20 | Dof zinc finger protein DOF1.3 | nucleus | KSSNNRFVPETYLNLQANPAAMARSMNF | 335 | 362 | 7.4e-05 | 1.000 | 0.41 |
| 21 | Calcium-dependent protein kinase 10 | nucleus | SSKPKKPNRDRKLNPFAGDFTRSPAPIR | 18 | 45 | 8.0e-05 | 1.000 | 0.637 |
| 22 | Protein STICHEL | nucleus | TRKPEMTNKSGHLNNIAGLNAETDVEVG | 1001 | 1028 | 8.1e-05 | 1.000 | 0.509 |
| 23 | Cyclin-dependent kinase inhibitor 3 | nucleoplasm nuclear part | RTRAAKTLALKRLNSSAADSALPNDSSC | 30 | 57 | 8.3e-05 | 1.000 | 0.401 |
| 24 | Transcription initiation factor TFIID subunit 12b | nuclear transcription factor complex nuclear part nucleus nucleoplasm part | AGLVGQSGHLPMLNGAAGAAQMNIQPQL | 232 | 259 | 8.8e-05 | 1.000 | 0.469 |
| 25 | Protein FAR-RED ELONGATED HYPOCOTYL 3 | nucleus | YGNQQTMQGLRQLNSIAPSYDSYYGPQQ | 774 | 801 | 8.8e-05 | 1.000 | 0.438 |
| 26 | SWI/SNF complex subunit SWI3C | nuclear part | TASPNVMFGNNQLNNPAAAGAASINQPS | 759 | 786 | 8.9e-05 | 1.000 | 0.478 |
| 27 | DNA (cytosine-5)-methyltransferase 4 | nucleus | KKKKKILQKGKNLNPRAGIAPVVSRMKA | 582 | 609 | 9.4e-05 | 1.000 | 0.403 |
| 28 | Protein NLP4 | nucleus | SNSSQCCSSETQLNSGATTDPPSTDVGG | 674 | 701 | 9.6e-05 | 1.000 | 0.597 |
| 29 | Squamosa promoter-binding-like protein 16 | nucleus | YASSGSDYSPPSLNSNAQERTGKISFKL | 450 | 477 | 9.8e-05 | 1.000 | 0.536 |
| 30 | Dof zinc finger protein DOF2.2 | nucleus | ETAAATTVDPSSLNGQAAERARLAKNSQ | 61 | 88 | 1.0e-04 | 1.000 | 0.655 |
| 31 | E3 ubiquitin ligase PQT3-like | nucleus | GSVIVKEATVSKLNTQAPKEEMQQQVAA | 445 | 472 | 1.1e-04 | 1.000 | 0.538 |
| 32 | Probable BOI-related E3 ubiquitin-protein ligase 3 | nucleus | SSRKRSREESVVLNPSAYMQIQKNPTDP | 95 | 122 | 1.1e-04 | 1.000 | 0.447 |
| 33 | Protein EMBRYONIC FLOWER 1 | nucleus | LSQASNNENTWNLNFVAANGKQKCGPNP | 801 | 828 | 1.1e-04 | 1.000 | 0.451 |
| 34 | DNA (cytosine-5)-methyltransferase 2 | nucleus | KRKKKILQKGKNLNPRAGLAHVVTRMKP | 579 | 606 | 1.1e-04 | 1.000 | 0.43 |
| 35 | VIN3-like protein 1 | nuclear part nuclear transcriptional repressor complex nucleus nucleoplasm part | PDSSGGEDNGVPLNSLAEADGGDHDDNC | 437 | 464 | 1.1e-04 | 1.000 | 0.699 |
| 36 | Ethylene-responsive transcription factor RAP2-12 | nucleus | YNNTEWDASLDFLNEDAVTTQDNGANPM | 315 | 342 | 1.2e-04 | 1.000 | 0.413 |
| 37 | Peptidyl-prolyl cis-trans isomerase FKBP53 | nucleus nuclear part | KNQKEKKKGENVLNEEAGQVQTGNVLKK | 269 | 296 | 1.2e-04 | 1.000 | 0.583 |
| 38 | Transcriptional corepressor LEUNIG | nucleus | GNPPQPQPQPQPLNQLALTNPQPQSSNH | 460 | 487 | 1.2e-04 | 1.000 | 0.907 |
| 39 | Splicing factor U2af large subunit A | nucleus nucleoplasm part nuclear part | AATLGPSQPSPHLNLAAVGLTPGASGGL | 328 | 355 | 1.2e-04 | 1.000 | 0.444 |
| 40 | Transcription factor bHLH74 | nucleus | LQSRDRNTPTLGLNPFAGFQGNIPNLSA | 293 | 320 | 1.2e-04 | 1.000 | 0.424 |
| 41 | Transcription factor PIF4 | nucleus | MSHDRSKNIEEKLNPNASSSSGGSSGCS | 179 | 206 | 1.2e-04 | 1.000 | 0.622 |
| 42 | Transcription factor bHLH148 | nucleus | SAKLFQALQQVRLNSSASTSSSPTAQKR | 58 | 85 | 1.2e-04 | 1.000 | 0.457 |
| 43 | AT-hook motif nuclear-localized protein 10 | nucleus | KYGPDSGEMSLGLNPGAPSFTVSQPSSG | 105 | 132 | 1.3e-04 | 1.000 | 0.732 |
| 44 | B3 domain-containing transcription factor VAL3 | nucleus | RNSSPLSRLHNDLNGGADSPFESKSRNV | 259 | 286 | 1.3e-04 | 1.000 | 0.516 |
| 45 | Transcriptional adapter ADA2a | nucleus | RKKEAEESMLLRLNHGAPGSIAGKTLKS | 412 | 439 | 1.3e-04 | 1.000 | 0.53 |
| 46 | Histone-lysine N-methyltransferase MEDEA | nuclear part nucleus | VATHASHHQSFDLNQPAAEDDNGGDNKS | 44 | 71 | 1.4e-04 | 1.000 | 0.565 |
| 47 | Serine/threonine-protein kinase KIPK2 | nucleus | EADRIKTLYRQVLNESAGKPGLPVDKGK | 154 | 181 | 1.4e-04 | 1.000 | 0.418 |
| 48 | Probable WRKY transcription factor 21 | nucleus | SDSLTLGTRSFSLNSNAKAPLLQLNQQT | 114 | 141 | 1.4e-04 | 1.000 | 0.49 |
| 49 | U-box domain-containing protein 40 | nucleus | LIQAIRDKPSVRLNHAATELDRRPNYFN | 161 | 188 | 1.5e-04 | 1.000 | 0.448 |
| 50 | Transcription initiation factor TFIID subunit 1b | nuclear transcription factor complex nuclear part nucleoplasm part | ENGDAGRENLKQLNSDARGRLSGLALQD | 385 | 412 | 1.5e-04 | 1.000 | 0.432 |
| 51 | Transcription factor bHLH123 | nucleus | AETSFGVMLQENLNLDATSNANANTTSS | 83 | 110 | 1.5e-04 | 1.000 | 0.483 |
| 52 | Squamosa promoter-binding-like protein 14 | nucleus | YASSGSDYSPPSLNSDAQDRTGKIVFKL | 485 | 512 | 1.6e-04 | 1.000 | 0.54 |
| 53 | DNA polymerase zeta catalytic subunit | nucleus | EIDLKPKGTFLNLNLQASVSQELSQISG | 979 | 1006 | 1.6e-04 | 1.000 | 0.431 |
| 54 | Splicing factor U2af large subunit B | nucleus nucleoplasm part nuclear part | AATLGPSQPNPNLNLGAVGLSSGSTGGL | 344 | 371 | 1.6e-04 | 1.000 | 0.468 |
| 55 | Transcription factor MYB106 | nucleus | LLHLQHYQNNNNLNKSAAPQQHCFTQKT | 169 | 196 | 1.6e-04 | 1.000 | 0.536 |
| 56 | Protein HAIKU1 | nucleus | RLQRIRPSPLTQLNRPAVPLPSMAPPQS | 86 | 113 | 1.7e-04 | 1.000 | 0.762 |
| 57 | B3 domain-containing transcription factor ABI3 | nucleus | QIGETCAAVAPQLNPVATTATGGTWMYW | 492 | 519 | 1.8e-04 | 1.000 | 0.432 |
| 58 | La-related protein 6B | nucleus | DPSLLRSLSLSRLNAGAPEFVPGRTTPP | 37 | 64 | 1.8e-04 | 1.000 | 0.566 |
| 59 | NAC domain-containing protein 43 | nucleus | LALPSVRSPYPSLNRSASYHAGLTQEYT | 310 | 337 | 1.8e-04 | 1.000 | 0.431 |
| 60 | VQ motif-containing protein 13 | nucleus | HSGPPEILTPTILNFPALDLSPDTPLMS | 108 | 135 | 1.8e-04 | 1.000 | 0.447 |
| 61 | YDG domain-containing protein At5g47150 | nucleus | PGCGTHIDVSSSLNHPAEKAFKHPRTGD | 16 | 43 | 1.8e-04 | 1.000 | 0.467 |
| 62 | Pre-mRNA-splicing factor SLU7-A | nuclear part | ASWGTDIPEDLELNEEALANALKKEDLS | 465 | 492 | 1.8e-04 | 1.000 | 0.483 |
| 63 | High mobility group B protein 15 | nucleus | TPQQSHGVLPNTLNISANPQGVAGGVTK | 218 | 245 | 1.9e-04 | 1.000 | 0.597 |
| 64 | Protein SMG7 | nuclear part nucleus | GKPEHLGSTGNGLNGPANFPFPGKQVPT | 988 | 1015 | 1.9e-04 | 1.000 | 0.602 |
| 65 | Pre-mRNA-splicing factor SLU7-B | nuclear part | ATWGTDIPEDLELNEEALANALKKEDLS | 465 | 492 | 1.9e-04 | 1.000 | 0.469 |
| 66 | CRC domain-containing protein TSO1 | nucleus | VKGEVVVPLVEDLNKEASLEDEEETSVE | 94 | 121 | 2.0e-04 | 1.000 | 0.527 |
| 67 | Polyadenylate-binding protein 5 | nucleus | EASAPQGIIPLPLNASANSHNAPQRSHK | 556 | 583 | 2.0e-04 | 1.000 | 0.623 |
| 68 | DEMETER-like protein 2 | nucleus | PWENKDVIPTIILNKEAGTSHDLVVNKE | 1082 | 1109 | 2.0e-04 | 1.000 | 0.402 |
| 69 | Zinc finger protein AZF1 | nucleus | EESAIGGHRGFDLNLPADQVSVTTS | 221 | 245 | 2.0e-04 | 1.000 | 0.552 |
| 70 | Regulatory protein NPR6 | nucleus | TSGSSLDSRLVYLNLGATNRDIGDDNSN | 415 | 442 | 2.0e-04 | 1.000 | 0.477 |
| 71 | Transcription factor MYB3R-3 | nucleus | VEEVVAASRMTSLNEYARSPQLPNPEPL | 296 | 323 | 2.1e-04 | 1.000 | 0.543 |
| 72 | Nuclear pore complex protein NUP214 (NUP214) | nuclear part | PFGKPLTSVKVDLNQAAPSTPSPSPGPT | 1306 | 1333 | 2.2e-04 | 1.000 | 0.483 |
| 73 | Synaptonemal complex protein 2 | nuclear part | ILNIREEHESKELNLKAKYDQELRQNQI | 622 | 649 | 2.2e-04 | 1.000 | 0.424 |
| 74 | DDT domain-containing protein DDR4 | nucleus | VEGVVGKRRYLDLNELAPVSGFDDGPST | 712 | 739 | 2.2e-04 | 1.000 | 0.5 |
| 75 | NAC domain containing protein 50 | nucleus | AEEDERPPSLCILNKEAPLPLLQYKRRR | 287 | 314 | 2.3e-04 | 1.000 | 0.58 |
| 76 | Transcription factor MYB3R-4 | nucleus | TFMQSNIDGNGCLNGQAENEIDSRQNSS | 211 | 238 | 2.3e-04 | 1.000 | 0.467 |
| 77 | Cyclin-T1-2 | nucleus | MDEALNENASGSESDASSVA | 1 | 20 | 2.3e-04 | 1.000 | 0.722 |
| 78 | Ethylene-responsive transcription factor ERF036 | nucleus | MTSLNSSASPTSSSSDQSD | 1 | 19 | 2.3e-04 | 1.000 | 0.794 |
| 79 | NAC domain containing protein 52 | nuclear part nucleus | EEEERPPRPVCVLNKEAPLPLLQYKRRR | 288 | 315 | 2.3e-04 | 1.000 | 0.594 |
| 80 | Regulatory protein NPR5 | nucleus | GSNNNLDSRLVYLNLGAGTGQMGPGRDQ | 431 | 458 | 2.3e-04 | 1.000 | 0.635 |
| 81 | Zinc finger CCCH domain-containing protein 5 | nuclear part | IAAKEREEAKAKLNDPAEQERLKAIEEE | 54 | 81 | 2.4e-04 | 1.000 | 0.53 |
| 82 | Protein CCA1 | nucleus | QQQEQRYPMALDLNFTAQLTPVDDQEEK | 518 | 545 | 2.4e-04 | 1.000 | 0.569 |
| 83 | Zinc finger protein ZAT9 | nucleus | HRNESVKQRMIDLNLPAPTEEDEVSVVF | 260 | 287 | 2.5e-04 | 1.000 | 0.535 |
| 84 | Probable NOT transcription complex subunit VIP2 | nucleus | MSNLHSSLNGSASNLPDGSGRSF | 1 | 23 | 2.5e-04 | 1.000 | 0.628 |
| 85 | Zinc finger protein ZAT4 | nucleus | EEEVSVKQRMIDLNLPAPNEEDETSLVF | 284 | 311 | 2.5e-04 | 1.000 | 0.43 |
| 86 | NAC domain-containing protein 7 | nucleus | QQHEHQQHMPYGLNASAYALNNPNLQCK | 202 | 229 | 2.6e-04 | 1.000 | 0.523 |
| 87 | 40S ribosomal protein S27-1 | nuclear part | MVLQNDIDLLNPPAELEKRKHKLKR | 1 | 25 | 2.6e-04 | 1.000 | 0.454 |
| 88 | Synaptonemal complex protein 2 | nuclear part | SANIGDLFSEGSLNPYADDPYAFD | 833 | 856 | 2.7e-04 | 1.000 | 0.528 |
| 89 | Synaptonemal complex protein 1 | nuclear part | SANIGDLFSEGSLNPYADDPYAFD | 848 | 871 | 2.7e-04 | 1.000 | 0.513 |
| 90 | Probable mediator of RNA polymerase II transcription subunit 19b | nucleoplasm part nuclear part | PFVLDELKEAFELNDTAPVELPPAEKGA | 85 | 112 | 2.7e-04 | 1.000 | 0.5 |
| 91 | Transcription factor bHLH143 | nucleus | LDTKQQKWLPLGLNPQACVQDKATEYFR | 3 | 30 | 2.7e-04 | 1.000 | 0.416 |
| 92 | Transcription repressor OFP10 | nucleus | MLNLQAKLNEKKVPLLT | 1 | 17 | 2.7e-04 | 1.000 | 0.479 |
| 93 | F-box protein KIB2 | nuclear part | MAPLNSQAAGEEESNYQCR | 1 | 19 | 2.7e-04 | 1.000 | 0.536 |
| 94 | Dof zinc finger protein DOF4.5 | nucleus | MDNLNVFANEDNQVNDVKP | 1 | 19 | 2.7e-04 | 1.000 | 0.718 |
| 95 | Dof zinc finger protein DOF4.4 | nucleus | MDNLNVFANEDNQVNGLKR | 1 | 19 | 2.7e-04 | 1.000 | 0.635 |
| 96 | Ethylene-responsive transcription factor RAP2-7 | nucleus | MLDLNLNADSPESTQYGGD | 1 | 19 | 2.7e-04 | 1.000 | 0.722 |
