# Supplementary material for: Evolution of A bHLH Interaction Motif
Source: Int J Mol Sci. 2021 Jan 5;22(1):447. doi: 10.3390/ijms22010447 (PMC7794824; doi:10.3390/ijms22010447)
Supplement: Supplementary file 1 [file ijms-22-00447-s001.zip › Table S2_chemical-shifts.docx]

**Supplemental Table 2.** Chemical shifts of MYB29-MIM peptide (top) and ASR3-MIM peptide (bottom).

| MYB29-MIM | | | | | |
| --- | --- | --- | --- | --- | --- |
| # | Residue | ^1^H^N^ (ppm) | ^15^N^H^ (ppm) | ^13^C^α^ (ppm) | ^13^C^β^ (ppm) |
| 184 | Ser | - | 122.06 | 58.48 | 63.73 |
| 185 | Ser | 8.74 | 118.31 | 58.79 | 63.61 |
| 186 | Thr | 8.33 | 115.81 | 62.31 | 69.53 |
| 187 | Ser | 8.37 | - | 58.92 | 63.91 |
| 188 | Lys | 8.47 | - | 56.73 | 32.75 |
| 189 | Leu | 8.20 | 122.45 | 55.39 | 42.12 |
| 190 | Leu | 8.24 | 122.60 | 55.27 | 42.15 |
| 191 | Asn | 8.42 | - | 53.33 | 38.77 |
| 192 | Lys | 8.37 | - | 56.73 | 32.99 |
| 193 | Val | 8.27 | 122.27 | 62.59 | 32.70 |
| 194 | Ala | 8.50 | 128.45 | 52.55 | 19.13 |
| 195 | Ala | 8.42 | 126.13 | 52.61 | 19.16 |
| 196 | Arg | 8.42 | - | 56.08 | 30.86 |
| 197 | Ala | 8.55 | - | 52.69 | 19.14 |
| 198 | Ser | 8.53 | 115.68 | 58.43 | 63.86 |
| 199 | Ser | 8.47 | - | 58.40 | 63.92 |

| ASR3-MIM | | | | | |
| --- | --- | --- | --- | --- | --- |
| # | Residue | ^1^H^N^ (ppm) | ^15^N^H^ (ppm) | ^13^C^α^ (ppm) | ^13^C^β^ (ppm) |
| 294 | Asp | - | 126.84 | 54.77 | 41.35 |
| 295 | Ser | 8.49 | 116.15 | 58.61 | 63.62 |
| 296 | Leu | 8.42 | 125.53 | 55.79 | 42.04 |
| 297 | Val | 8.04 | 120.78 | 62.98 | 32.55 |
| 298 | Ala | - | - | 53.11 | 18.98 |
| 299 | Val | 8.22 | 120.50 | 63.02 | 32.57 |
| 300 | Leu | 8.33 | - | 55.70 | 42.02 |
| 301 | Asn | 8.55 | 120.09 | 53.52 | 38.63 |
| 302 | Lys | 8.34 | - | 57.12 | - |
| 303 | Leu | - | - | 55.71 | 42.17 |
| 304 | Ala | 8.27 | 124.13 | 53.14 | 18.98 |
| 305 | Asp | 8.31 | 119.73 | 54.92 | 41.03 |
| 306 | Ala | 8.18 | 123.78 | 52.97 | 18.98 |
| 307 | Val | 8.13 | 119.70 | 63.00 | 32.57 |
| 308 | Ala | 8.39 | 127.26 | 53.01 | 18.98 |
| 309 | Lys | - | - | 56.40 | - |
